# Small sight—Big might: Economic impact of bird tourism shows opportunities for rural communities and biodiversity conservation

**DOI:** 10.1371/journal.pone.0268594

**Published:** 2022-07-06

**Authors:** Tobias Schwoerer, Natalie G. Dawson

**Affiliations:** 1 International Arctic Research Center, University of Alaska Fairbanks, Fairbanks, Alaska, United States of America; 2 National Audubon Society, Anchorage, Alaska, United States of America; 3 Department of Environmental Science, Alaska Pacific University, Anchorage, Alaska, United States of America; 4 Caurina Consulting LLC, Haines, Alaska, United States of America; University of Brasilia, BRAZIL

## Abstract

Birdwatching is considered one of the fastest growing nature-based tourism sectors in the world. Tourists who identify as birdwatchers tend to be well-educated and wealthy travellers with a specific interest in the places they visit. Birdwatchers can bring economic resources to remote communities diversifying their economies and contribute to biodiversity conservation in areas of bird habitat with global significance. Alaska plays a critical role in understanding the link between bird conservation and bird tourism as it supports the world’s largest concentration of shorebirds and is a global breeding hotspot for hundreds of migratory species, including many species of conservation concern for their decline across their ranges. Alaska is also a global destination for birders due to the large congregations of birds that occur during the spring, summer and fall seasons. Despite its global importance, relatively little information exists on the significance of bird tourism in Alaska or on opportunities for community development that align with conservation. This study used ebird data to look at trends in Alaska birdwatching and applied existing information from the Alaska Visitor Statistics Program to estimate visitor expenditures and the impact of that spending on Alaska’s regional economies. In 2016, nearly 300,000 birdwatchers visited Alaska and spent $378 million, supporting approximately 4,000 jobs. The study describes bird tourism’s contributions to local jobs and income in remote rural and urban economies and discusses opportunities for developing and expanding the nature-based tourism sector. The study points toward the importance of partnering with rural communities and landowners to advance both economic opportunities and biodiversity conservation actions. The need for new data collection addressing niche market development and economic diversification is also discussed.

## Introduction

Participation in U.S. nature-based outdoor recreation has increased over the past decade, continuing a long-term, upward trend [1]. Wildlife-related activities, especially wildlife viewing have increased by 20% between 2011 and 2016, outpacing participation in hunting and fishing [2]. Wildlife-related tourism can provide direct benefits to wildlife and wildlife conservation efforts if tourists engage as conservation partners during their activities within a given place [3]. As the conservation of biological diversity becomes a global priority in the face of climate change, opportunities exist to highlight the relationship between wildlife-related tourism, economic diversification for communities, and conservation action.

Among increasing wildlife-related tourists in the U.S., birdwatchers, also called birders, have seen steady increases in participation and are considered the world’s largest group of “eco-tourists” [4]. Birdwatchers can also include local residents within a community. Birding can be defined as “observation, identification, and photography of birds for recreational purposes” [5]. Birding tourism, like many wildlife tourism activities, requires interaction between a visitor and a local community and depends on the ecological integrity of a specific location to support the bird species that draw tourists to a particular place. Many of the places that draw birding tourism are also remote, requiring significant expenditures to access. Studies show that birding tourists tend to be well-educated, wealthy, and committed to their chosen tourism activity [6].

The growth and popularity of birding has provided new revenue to rural communities, regions, and countries [6,7]. Expenses related to wildlife viewing in the U.S. increased 29% from 2011 to 2018 [2]. In 2016, there were 45 million birders in the U.S. spending $39 billion on travel and equipment aimed at observing birds [6]. Thus, tourism experiences can be enhanced by the conservation activities within a given community that in turn strengthens a model of economic development based on ecological integrity. Growth in wildlife viewing is also happening at a time of unprecedented declines in global biodiversity [8]. Effective and immediate conservation action at a global scale is required to reduce and halt the decline [8,9].

Alaska, is one of the few remaining storehouses of intact habitat and complete wildlife assemblages. Thus, Alaska plays a central role in global conservation action [9]. It is home to the world’s largest concentration of shore birds and is a globally significant breeding hotspot for migratory birds. Alaska supports over one third of the world’s shorebird populations some of which breed nowhere else and are threatened by habitat loss and climate change [10].

Alaska’s high-quality bird habitat provides unmatched viewing opportunities for rare and threatened species. Over 550 species of birds have been documented across Alaska’s vast landscape [11,12]. Alaska also holds the most globally-significant Important Bird Areas (IBAs) of any U.S. state. Many of Alaska’s Important Bird Areas do not have conservation status and thus are not protected [13]. Yet, IBAs are essential places or habitat for birds’ nesting, foraging, and migrating [14].

While birds can be observed in Alaska year-round, the best birding season extends from May until September, overlapping with the presence of migratory birds for which Alaska serves as a globally important migratory stop-over and breeding ground [11,15]. Most birdwatchers visit Alaska in June followed by July and August [16]. Between 2001 and 2019, the number of users of ebird, a citizen science bird observation app, increased nine-fold for submissions in Alaska [17]. Similarly, the reported average birding effort per birder has increased from five submissions per birder in 2008 to over 40 submissions per birder in 2020 [17].

Despite increasing birdwatching trends in Alaska, relatively little is known about bird tourism’s influence on Alaska’s regional economies. Such information informs policy supporting bird habitat conservation and sustainable economic development. Nature-based tourism is often seen as a tool for habitat conservation in regions that are economically disadvantaged and where investments into e.g. local transportation infrastructure can both serve local communities and a growing visitor industry [18]. Given immediate global habitat conservation needs, Alaska can diversify local economies and attract new revenue for the remote regions of the state that remain underserved and predominately inhabited by the state’s rural Indigenous Alaska Native peoples [19].

Only one of two recent Alaska visitor surveys asked respondents about bird watching [20,21]. In a survey related to visitor transportation, the second most reported outdoor activity was wildlife viewing which included bird watching, with 77% of visitors reporting participation. The Alaska Visitor Statistics Program found that 9% of visitors in 2016 engaged in bird watching [21].

The past two decades saw little attention towards monitoring tourism trends across the Circumpolar North, thus few information sources exist on the importance of bird tourism to local economies [22]. Tourism research in Alaska, for example, has focused on the more populated parts of the state with small sample sizes and statistical challenges for informing the remote rural parts of Alaska [22,23]. These parts of Alaska are also the most likely to face the greatest vulnerabilities through potential negative impacts from large-scale economic development and are in need of sustainable economic development opportunities that meet community needs [22]. One of those community needs is retaining the ecological integrity of surrounding lands and waters that support cultural and traditional subsistence practices that to this day form the backbone of local economies [24].

This study contributes economic information about bird tourism in Alaska describing the regional economic impact of bird tourism across Alaska’s ecologically defined Bird Conservation Regions [15]. We discuss economic impacts on both remote rural and urban economies and opportunities for expanding nature-based tourism in regions with less diverse economies. The study also points towards Alaska’s role in global efforts to reduce biodiversity loss and the important role rural communities can play for positive outcomes in conservation and community development.

### Alaska’s bird conservation regions

**Fig 1:**
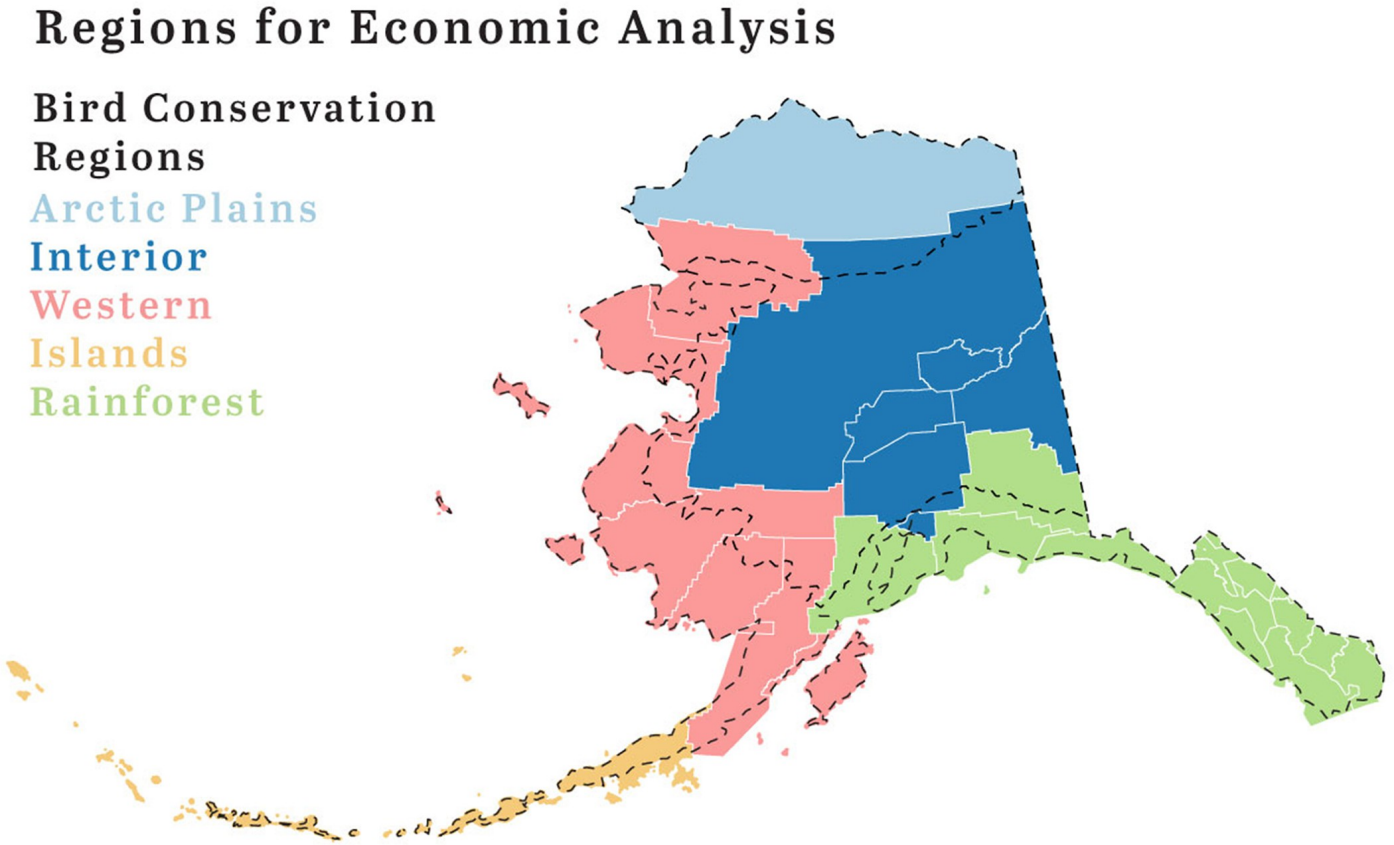
NABCI bird conservation regions used for economic analysis with borough and census area outlines. Reprinted from U.S. NABCI (2020) under a CC BY license, with permission from NABCI, original copyright 2021.

The North American Bird Conservation Initiative divides Alaska into five conservation regions (Fig 1) [15]. First, the Northern Pacific Rainforest Bird Conservation Region we call *Rainforest region*. It encompasses all of Southeast Alaska and the Gulf of Alaska coast. This region is largely roadless with communities connected through the Alaska Marine Highway System, the state-owned ferries. The region’s forests, offshore islands, estuarine and freshwater wetlands, and rocky shorelines are a critical breeding habitat and a migration stop over for thousands of migratory bird species. Migration stopovers in estuaries along the Southeast Alaska coastline including Prince William Sound boast some of the highest numbers of shorebirds in the state of Alaska. The region’s basic sector industries include tourism, seafood, timber, and mining [25].

**Fig 2:**
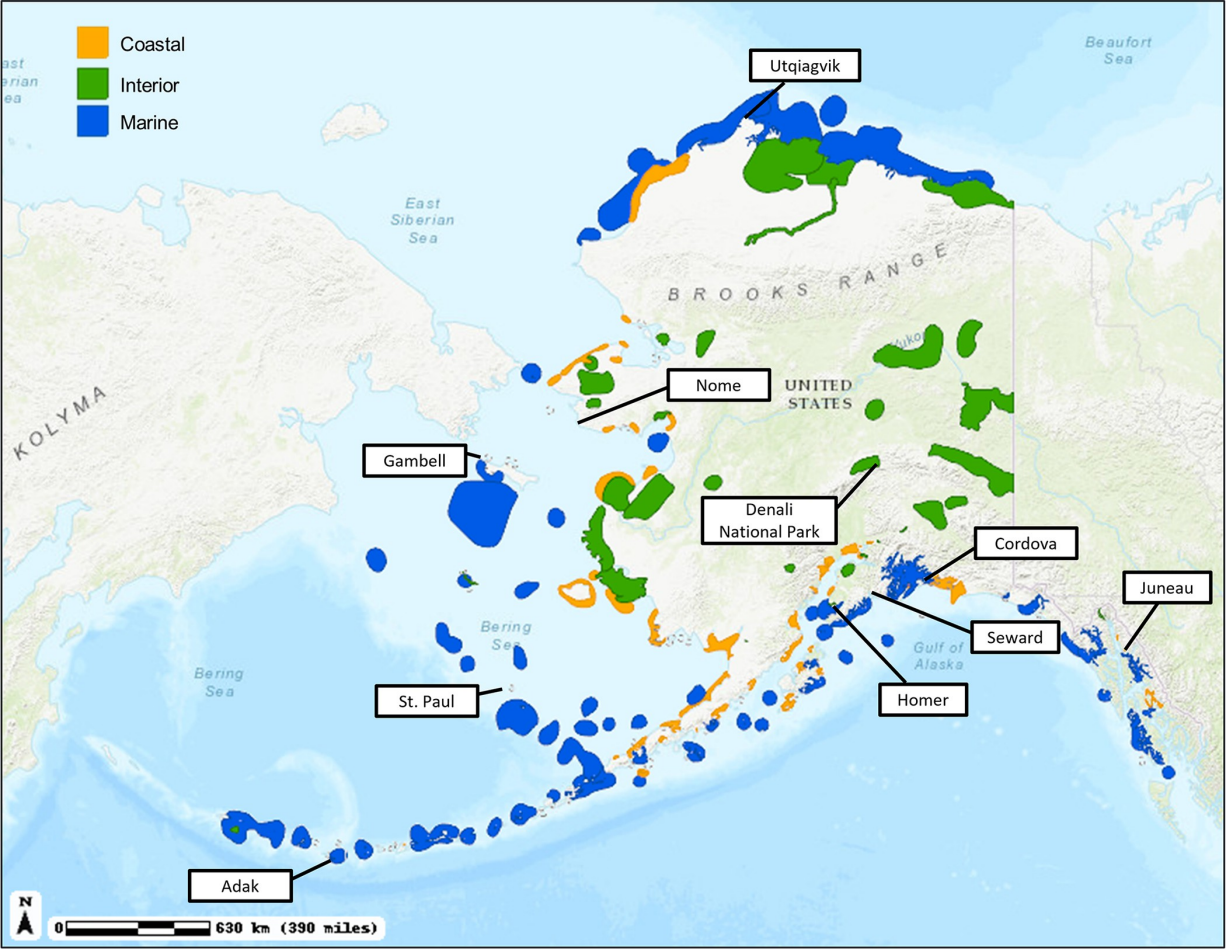
Alaska’s Important Bird Areas (IBAs) and communities that are birdwatching hotspots.

This region is known for its shorebird festivals occurring annually each spring in Homer, Yakutat, and Cordova, birdwatching hotspots that are close to rural communities (Fig 2). The festivals attract both Alaska resident birders and birders from outside of Alaska celebrating the seasonal congregations of shorebirds in this region. Many of the migratory and resident bird species have high conservation status and important cultural significance, such as the Snow Goose (*Anser caerulescens*), Marbled Murrelet (*Brachyramphus marmoratus*), Chestnut backed Chickadee (*Poecile rufescens*), and Red-breasted Sapsucker (*Sphyrapicus ruber*). Black Oystercatchers (*Haematopus bachmani*), Rock Sandpipers (*Calidris ptilocnemis*), Black Turnstones (*Arenaria melanocephala*), and Surfbirds (*Calidris virgate*) are common wintering species. The region’s pelagic waters support large populations of shearwaters, storm-petrels, alcids, and Black-footed Albatross (*Phoebastria nigripes*) [15].

The Northwestern Interior Forest Bird Conservation Region we call *Interior region*. The forested lowlands and uplands of the Interior region are breeding grounds for shorebirds like the Greater (*Tringa melanoleuca*) and Lesser Yellowlegs (*Tringa flavipes*), Solitary (*Tringa solitaria*), and Spotted Sandpipers (*Actitis macularius*). Alpine habitats support American Golden-Plovers (*Pluvialis dominica*) and Surfbirds (*Calidris virgate*) [15]. A large part of this region is also known as Alaska’s Railbelt region which is the only region in Alaska that is connected by roads providing the transportation infrastructure for accessing Important Bird Areas such as Denali National Park and Preserve (Fig 2). It also forms the state’s population and economic center with Alaska’s largest cities Anchorage and Fairbanks and has the most diversified economy including oil and gas, tourism, mining, and transportation [26].

Further, the Arctic Plains and Mountains Bird Conservation Region we call *Arctic Plains*. This region is dominated by waterfowl and shorebirds where the most abundant species along the coastal plain include Northern Pintails (*Anas acuta*), King Eiders (*Somateria spectabilis*), Long-tailed Duck (*Clangula hyemalis*), Sandpipers, and Red-necked Phalarope (*Phalaropus lobatus*) [15]. Communities in this region are not connected to roads and local economies are mixed cash-subsistence economies. This region includes Utqiaġvik, a known birding destination for the large congregations of eiders that occur in the region (Fig 2). The region also includes Alaska’s North Slope where most of Alaska’s petroleum resources are being developed along the coastal plain overlapping with Important Bird Areas (Fig 2). The oil and gas industry includes North America’s largest oil field, the Prudhoe Bay oil fields which are connected to Alaska’s Railbelt by the Dalton Highway [27].

The fourth region is the Western Alaska Bird Conservation Region which we refer to *Western Alaska*. It includes all of western coastal Alaska starting with the Northwest Arctic Borough to the North and ending with the Bristol Bay Borough and Kodiak to the South (Fig 1) [15]. High densities of breeding waterfowl and shorebirds are common especially during migration including Dunlin (*Calidris alpine*), Western Sandpiper (*Calidris mauri*), Red Knot (*Calidris canutus*), and Bar-tailed Godwit (*Limosa lapponica*). Wintering sea ducks such as Steller’s Eider (*Polysticta stelleri*), Harlequin Duck (*Histrionicus histrionicus*), Surf Scoter (*Melanitta perspicillata*), and Black Scoter (*Melanitta Americana*) can be found along the Alaska Peninsula coast. This region is also roadless and supports the world’s largest wild commercial salmon fishery in Bristol Bay [28]. Bristol Bay, the Yukon-Kuskokwim Delta, and Safety Sound near Nome host some of the largest congregations of waterfowl in North America, including the only nesting and breeding sites for the Emperor Goose (*Anser canagicus*), an important species for subsistence food and also a species of conservation concern due to declining populations in the face of climate change. Nome and Gambell, a community on St. Lawrence Island in the Bering Straits are the region’s birding hotspots (Fig 2). The region’s mixed cash-subsistence economy is supported by commercial fishing [24,29].

The fifth region is the Aleutian / Bering Sea Islands Bird Conservation Region, which we call *Islands* region. Seabirds dominate this region with some exclusively breeding here including Red-legged Kittiwake (*Rissa brevirostris*), Least Auklet (*Aethia pusilla*), and Whiskered Auklet (*Aethia pygmaea*) [15]. The Pribilof Islands contain the largest nesting colonies of seabirds along the continental shelf of Alaska. They are an important destination for remote bird watching with St. Paul Island being the number one Alaska hotspot with over 300 bird species that have been documented (Fig 2) [30]. The International Port of Dutch Harbor in the City of Unalaska is the largest fisheries port in the U.S. by volume caught, emphasizing the importance of commercial fisheries to the region besides small-scale mixed cash-subsistence economies [31].

Since the launch of Alaska ebird in May 2007, about 12,000 ebird app users reported over 358,000 bird observations [12]. Table 1 illustrates that the remote regions of Alaska have higher bird diversity indicated by the number of observable species in the Western and Islands regions while the road-connected Interior region shows higher birding effort from Alaska residents and visitors combined [12]. More detailed ebird data by census area can be viewed in S1 Table.

**Table 1:** Observed number of species and ebird submissions by Alaska Bird Conservation Region, 2007–2021.

Bird Conservation Region	Species	Submissions
Islands region	367	20,200
Western region	324	55,611
Interior region	281	122,492
Rainforest region	278	142,709
Arctic Plains	278	17,149
Total	556	358,161

Source: Alaska ebird data explorer [12].

## Methods

### Data

The Alaska Department of Commerce, Community, and Economic Development and the Alaska Travel Industry Association partner in the Alaska Visitor Statistics Program (AVSP). The program monitors annual visitor volume using several data sources including border crossings, and airline travel. In addition, AVSP conducts a visitor survey every five years, collecting data on activities, demographics, expenditures, opinions and trip planning through intercept surveys when visitors leave Alaska at airports, ferry terminals, cruise ship docks and other exit points [21]. The data is publicly available [32].

We also used data on bird observations reported in ebird, a citizen science bird observation platform managed by the Cornell Lab of Ornithology. With data from 2007 to 2021 we calculated bird watching effort as an additional metric to the AVSP data [16]. Each record in ebird corresponds to an observed bird species and includes information on the date of the observation and the group size that the reporting bird observer was part of. For future reference, we call these birders who submit bird observations via the ebird app “*reporting bird observers*.”

### Birder spending

The 2016 AVSP survey included 5,147 intercept in-person interviews and 779 online surveys for a total of 5,926 respondents whose answers were then scaled to the population of visitors following AVSP methodology [21]. We used R for data management and survey data analysis to first subset the data into visitors who reported to have been bird watching on their Alaska trip (S1 Code). We scaled expenditures to the population level by applying market shares according to the main purpose of the trip including visiting for pleasure/vacation, visiting friends, and business travel [21]. Specifically, we calculated the scale factors to equal 438 for vacationers, 267 for visiting friends, and 278 for business travellers (S1 Code).

AVSP’s expenditure data includes spending by category and by location which we used to attribute expenditures to economic sectors and regions (S2 Table). This attribution is especially important for tourism analysis as the sector is comprised of many sub-sectors including lodging; tours, activities, and entertainment; gifts, souvenirs, and clothing; food and beverages; and rental cars, fuel, and other transportation [21]. AVSP includes expenditure category “other”, which we proportionally re-allocated across the remaining five spending categories (S1 and S2 Tables).

For the purpose of regional economic analysis, we aggregated AVSP expenditures into regional economies consisting of groups of Alaska’s organized boroughs and census areas (Fig 1). These regions roughly encompassed Alaska’s five Bird Conservation Regions as defined by the North American Bird Conservation Initiative and described in more detail in the following section [15] (Fig 1 and S1 Table). The aggregation allowed for a region-specific analysis of economic impacts related to each Bird Conservation Region.

To analyze the potential discrepancy between where expenditures occur and where birds are being observed and to validate the AVSP data, we used eBird data to calculate the average group size per *reporting bird observer* across submitted observations per region [16]. If observers did not report group size, we set group size equal to one. We then summed the average group sizes for all *reporting bird observers* to estimate the number of observers for each of Alaska’s five Bird Conservation Regions (S1 Code) (Cornell Lab of Ornithology, 2021).

### Input-output analysis

We used input-output analysis to investigate the economic impacts of bird tourism [33,34], using IMPLAN software, a widely used input-output model that was developed by the U.S. Forest Service [35]. Our analysis measured the *net* changes to a regional economy that are attributable to related bird-watching visitor spending.

The five regional economic models followed roughly the outlined Alaska Bird Conservation Regions and each consisted of a subset of Alaska’s census areas and boroughs (Fig 1) [15] (S2 Table). IMPLAN simulates interactions of 528 unique economic sectors, using data collected by federal and state government agencies. For each AVSP expenditure category we assigned the most closely related IMPLAN sector among IMPLAN’s industry and commodity accounts and then applied the estimated total expenditures to those accounts (S3 Table).

For each region, we paid particular attention to IMPLAN’s industry and commodity accounts that best described the expenditure category by region. For example, for lodging in the Arctic Plains and Western Alaska regions, we assigned lodging expenditures as an industry change equally proportioned among the hotels/motels and other accommodations accounts, whereas for the Interior and Rainforest regions we used a ¾ and ¼ allocation respectively (S3 Table) [33]. This approach acknowledged the relatively small number of hotels in the Arctic Plains and Western Alaska regions compared to other accommodations such as e.g. bed and breakfasts [36]. Similarly, we modelled expenditures on tours as a commodity change and assigned it to the scenic and sightseeing sector in the Arctic Plains and Western Alaska regions while also using the museums and other amusement accounts for the Interior region, consistent with a more diverse supply of entertainment services in the Interior region (S3 Table) [36].

We measured the economic impact of bird watching through the following effects [33]. ***Direct effects***: These effects are a result of actual bird watching expenditures. For example, a visitor purchasing a $200 bird watch tour would result in a $200 direct effect of bird watching. Besides direct effects there are additional impacts that occur through economic activity generated by the initial direct expenditures. These additional impacts are also known as multiplier effects and include the following. ***Indirect effects***: These effects arise due to linkages in the supply chain, such as lodges purchasing goods and services from local suppliers, buying inventory, paying rent, or hiring service personnel. ***Induced effects***: These effects are a result of employee household spending. For example, when a birdwatcher purchases binoculars at a local outdoor store, some portion of that dollar amount goes toward paying the wages of the sales personnel, who then re-circulate earned wages in other ways such as purchases at a local grocery. For any retail accounts presented in our modelling, we had IMPLAN apply the retailers’ margin to visitor spending in retail stores, to correctly estimate the economic impacts in the retail sector (French, 2019).

The analysis quantified economic impacts of bird watching using the following metrics: ***Employment*** (jobs), which IMPLAN defines as the total number of all wage and salary employees and self-employed jobs in a region. This job count includes both full-time and part-time workers and can be considered as the annual average job count. It is comparable to employment numbers reported by the U.S. Bureau of Labor Statistics and the Alaska Department of Labor and Workforce Development (ADLWD) [35,37,38]. Thus, one job lasting 12 months equal two jobs lasting six months or three jobs lasting four months, where a job can be either full time or part time. Since a person can hold more than one job, the job count is not necessarily the same as the count of employed persons. ***Labor Income*** includes all forms of employment income, wages, benefits and proprietor income. The latter is important in the context of bird watching as many tour operators are independent sole proprietors. ***Value Added*** is another common measure of impact which includes labor income and payment to business owners, investors, landlords and government. It is analogous to Gross Domestic Product (GDP). Finally, we present economic model results in the form of ***Output*** which is a measure of the value of production in a calendar year, associated with all economic activity directly or indirectly related to bird watching expenditures. The IMPLAN event year was set to 2016 to be consistent with the most recent AVSP data collected in 2016. Output is commonly also referred to as annual revenue plus net inventory change [39]. All dollar values related to the presented economic impacts are in 2021 US dollars, adjusted for inflation [35]. We also used IMPLAN to estimate the generated state and local tax contributions associated with birdwatchers’ expenditures [35]. Finally, we used IMPLAN to investigate the bird tourism sector’s contributions to the local, state, and federal tax base [35].

Besides the economic impact analysis, we further investigated differences between bird-watching visitors and visitors who did not engage in bird watching using R’s survey analysis package [40] (S1 Code).

## Results

### Birder spending and characteristics

In 2016, Alaska received an estimated 294,500 visitors who indicated to have watched birds during their visit to Alaska. This estimate compares to the only historically available estimate of 157,290 in 2001 [7], suggesting a doubling of the bird watching niche market within this time period.

On average, birders spent approximately 56% more per person than visitors who did not engage in bird watching, yet birdwatchers stated 9% lower household income compared to other visitors, likely related to the disproportionate number of retirees (54%) among birdwatchers versus other visitors (45%) (Table 2). Also, birdwatchers tend to stay longer and are more active during their trips. On average, birders spent four more nights and engaged in twice as many activities than non-birders (Table 2). Higher than average educational attainment and the tendency of birders to be middle-age or older was reflected in AVSP and in a nationwide survey of birders, suggesting that the limited data we used was representative of birdwatchers in general [6].

**Table 2:** AVSP survey respondent characteristics, 2016.

	Birdwatchers, n = 743		Other visitors, n = 5,174	
Variable	Mean	SD	Mean	SD
Per person expenditures, US$ ^a^	1,694	2,780	1,084	1,746
Household income before taxes, US$ ^a^	102,170	48,263	110,690	52,782
Nights spent on most recent trip	13.0	10.3	8.9	8.8
Number of local activities	5.9	2.5	3.4	2.3
Number of previous Alaska vacations	1.1	0.3	1.1	0.4
Group size	2.1	1.5	2.3	1.6

^a^2016 U.S. dollars, statistics calculated from mid-points in reported income intervals (S5 Table).

In 2016, four in every five birdwatchers arrived in Alaska independently (not on cruise ships), illustrating that most birdwatchers are independent travellers, reaching Alaska on various transportation modes, other than cruise ships. Independent travellers are also more likely to reach remote rural areas compared to other visitors [7,41].

In 2016, the estimated Alaska bird tourism expenditures amounted to $378 million in 2016 U.S. dollars with the majority (28%) of total spending going towards tours and activities, followed by lodging (22%), food and beverages (20%), gifts and souvenirs (16%) and transportation (15%) (Table 3).

**Table 3:** Alaska bird tourism expenditures by category in 2016 $ million and bird watch effort by Bird Conservation Region.

Category	Arctic	Islands	Interior	Rainforest	Western	Total
Lodging	0.8	n/a	42.8	34.2	0.2	83.0
Tours, activities, entertainment	0.2	n/a	25.0	71.5	7.4	104.1
Gifts, souvenirs, clothing	0.2	n/a	20.7	36.3	3.5	60.7
Food and beverages	0.3	n/a	37.9	31.2	5.4	74.8
Rental cars, fuel, transportation	0.3	n/a	40.2	11.0	3.9	55.4
Total spending^a^	1.8	n/a	166.5	184.2	25.4	377.9
% total spending	<1%	n/a	44%	49%	7%	
Estimated 2016 birdwatchers ^b^	5,890	n/a	220,875	253,270	14,725	294,500
% total birdwatchers	<2%	n/a	75%	86%	<5%	
ebird reporting bird observers ^c^	604	985	4,322	4,312	1,602	11,825
% total ebird rep. bird observer	5%	8%	37%	36%	14%	
Mean rep. bird observer group size	2.6	4.9	2.9	2.7	3.6	3.0

^a^Source: Alaska Visitor Statistics Program VII [21].

^b^Estimated birdwatcher volume by region, based on n = 606 AVSP respondents indicating regions visited [21].

^c^For regional comparison only. Ebird observers calculated from the average observer group sizes as reported through ebird using most recently available data from eBird Basic Dataset [16].

Among Alaska’s Bird Conservation Regions, the Rainforest region received the most birders and had the largest spending with $184.2 million, almost half of the statewide total. Interesting to note, birdwatchers spent more on tours and activities in this region than in any other Alaska region, amounting to $71 million or 39% of the regional total. The bulk of birdwatching tours in this region are one-day trips related to the cruise ship industry.

Western Alaska also has a high proportion of spending in the tours and activities category, indicating that these two regions may have relatively well-developed bird tourism sectors compared to the other regions in Alaska. Nome has been a known birding destination since the mid 1980’s and independent birding travel is relatively easy due to a small road network and Nome being a larger community with existing infrastructure that can support a growing birding industry such as lodging. Also, with an increasingly ice-free Bering Strait in summer months, the community is seeing increased visitation by cruise ships [42].

The Interior region’s bird tourism sector is similar in size to the sector in the Rainforest region both based on the estimated spending and the number of reporting bird observers estimated using ebird data. Birders visiting the Interior spent most on lodging (26%) and less on tours and activities, only a third of what birders spent on tours and activities in the Rainforest region.

Birders visiting the Arctic Plains region had a similar spending pattern to those visiting the Interior. Most birding in the Arctic Plains is accessed via the Dalton Highway, thus individual transportation by car may result in smaller group sizes and a more individualized birding experience in the Arctic Plains and Interior regions (Table 3).

Unfortunately, no data were available for the Islands region but ebird data suggests that the bird tourism sector in this region is likely twice the size of the Arctic Plains region’s birding sector (Table 3). The Island region also has the largest birder group size showing that birding is relatively organized. The primary reason for this fact is the daunting logistics and expense to travel to this very remote region of Alaska where air transportation is also highly dependent on weather. Group travel is therefore an advantage when traveling to areas like Gambell on St. Lawrence Island, Adak in the Aleutian Chain, or St. Paul in the Pribilof Islands (Fig 2). According to several sources, ornithologists rank the Pribilof Islands as one of the world’s premier birding locations, and because of this birding attention, these islands have been referred as “The Galapagos of the North” [43]. Due to data limitations described earlier, little information is available on how much economic impact bird tourism generates in this region (Table 3).

Our results also underline the limitations of AVSP data and tourism surveys that are not targeting certain segments of the market such as independent travellers. Most travellers to the Islands region are considered independent travellers even though they may be joining organized trips with local guides.

### Economic impacts

A total of 3,273 annual average statewide jobs are directly associated with the expenditures of bird tourism and an additional 1,100 jobs stem from businesses supporting and supplying goods and services to the bird tourism sector (Table 4). The direct employment alone compares to the direct employment of Alaska’s mining industry, which in 2020 had an annual average job count of 3,111 or Alaska’s telecommunications sector with 3,500 direct jobs [37]. Half of Alaska’s total jobs that are linked to bird tourism are associated with the Interior region’s bird tourism sector, followed by the Rainforest region (44%), Western Alaska (6%) and Arctic Plains (<1%) (Table 5).

**Table 4:** Economic impacts of bird tourism in Alaska, 2021 US$ million.

Impact type	Employment	Labor income	Value added	Output
Direct effect	3,273	$141.6	$214.7	$383.0
Indirect effect	492	$30.7	$52.7	$103.0
Induced effect	613	$31.6	$59.1	$98.2
Total	4,378	$203.9	$326.5	$584.2

**Table 5:** Total economic impact of bird tourism by Alaska Bird Conservation Region and size of regional economies, 2021 US$ million.

Total impact ^a^	Arctic	Interior	Rainforest	Western
Employment	15	1,919	1,696	226
Labor income	0.7	89.5	77.8	11.7
Value added	1.2	150.8	117.7	18.6
Output	2.2	271.1	210.7	33.9
**Gross Regional Product** ^b^	2,000.1	28,000.0	6,000.5	2,000.5

^a^For detailed direct, indirect, and induced effects (S4 Table). No data were available for the Islands region. All US$ amounts shown in 2021$ adjusted for inflation.

^b^Note, to be compared with the “value added” row to get the relative size of the bird tourism sector in the overall regional economy. Source: IMPLAN Group.

The majority (1,079) of the 4,378 total jobs generated by bird tourism were in the food services sector followed by 700 annual average jobs in the scenic and sightseeing transportation and support services sector (Table 6). Jobs in the lodging and retail sectors are also among the top-ranking employment generators yet show much lower effects on labor income due to the lower wages in these sectors. A national survey of birdwatchers found similar economic impacts across these sectors, underlining the significance and representativeness of the results [6].

**Table 6:** Total economic impact by sector ranked by employment impact, 2021 US$ million.

Sector	Employment	Labor Income	Value added	Output
Food services	1,079	35.8	50.8	93.8
Scenic and sightseeing transportation and support	702	46.6	49.3	82.7
Hotels and motels	552	19.7	35.2	74.5
Retail Stores—Miscellaneous	209	6.1	9.1	9.5
Automotive equipment rental	195	8.9	28.1	45.2
Recreation industries	185	4.4	6.8	12.6
Sporting goods, etc. retail	170	5.0	8.2	8.4
Other accommodations	151	7.7	13.1	24.3
General merchandise retail	105	4.6	7.4	7.6
Real estate	78	1.5	11.3	17.4

The bird tourism sector also generated a total of $204 million in wages, salary, and benefits associated with the forementioned jobs (Table 4). Its contribution to Alaska’s GDP as measured through value added, amounted to $326 million, roughly 1% of Alaska’s current-dollar GDP (Table 5). This result is consistent with the larger outdoor industry contributing 3.9% to the overall Alaska economy, twice the national average and underlining that Alaska compared to other U.S. states relies more on the outdoor recreation industry [44,45]. Regionally, the bird tourism sector contributes most to the regional economy in the Rainforest region (2%) followed by Western Alaska (1%), Interior (0.5%) and Arctic Plains (<0.1%). This result further supports the previously mentioned regional differences and highlights opportunities for growth in Western Alaska and the Arctic Plains region (Table 5).

The total value of all economic activity associated with Alaska’s bird tourism sector amounted to $584 million (Table 4), roughly equivalent to the five-year annual average harvest value fishers receive for commercially caught salmon in Alaska which between 2015 and 2020 equalled $607 million [46].

Bird tourism also generated $47.8 million in state and local tax contributions in form of corporate dividends, social insurance, excise taxes, local sales and property taxes, severance taxes, corporate profits, fines and fees, and motor vehicle registrations. In addition, federal tax contributions including social insurance, excise taxes, customs duty, corporate profits tax and personal income taxes amounted to $39.5 million.

## Discussion

This study examined location-based tourism expenditures and used input-output modelling to demonstrate the economic impacts of bird tourism on Alaska’s economy. The study provides a one-year snapshot about employment and income generated by bird tourism and shows how this economic impact varied across Alaska regions. Thus, the approach is different from ecosystem service valuation that estimates net economic value changes [47]. Nature-based tourism is a growing economic sector that contributes to economic diversification [22,45,48]. Research continues to suggest that new tourism development, if designed with community insights, can also become sustainable and relevant in new ways within communities where tourism brings visitors and economic benefits [49].

We find the largest opportunities for sustainable economic growth through the development of nature-based tourism in the remote and underserved parts of Alaska. In regions with higher poverty rates, niche market development can play an important role for growing and diversifying the economy. Bird tourism results in either new revenues to the region that otherwise would not have occurred, or revenues that in the absence of bird habitat conservation and associated visitation of healthy bird populations, would be lost. Especially in the remote regions, bird tourism provides critical cash flow and rare employment and income opportunities for local communities interested in diversifying their local economies [36]. Our results illustrate that bird tourism brings substantial amounts of new money to Alaska’s economy, providing jobs, income, and government revenue.

The presented economic impacts are likely a lower bound to the true economic impacts of bird tourism in Alaska. Part of the reason for this is the way in which tourism data are collected [23]. For example, the impacts of the COVID-19 pandemic halted most industrial-scale tourism in Alaska driven by the cruise ship industry, but independent travellers found ways to bolster tourism in some regions of the state [50]. Our results indicate that current tourism data collection efforts are particularly challenging for quantifying birding tourism because many Alaska birding destinations are not accessible by cruise ship and instead are more likely accessed by independent visitors.

Historically, most studies of Alaska tourism relied on AVSP, designed to survey the cruise-ship-related portion of Alaska visitors [51–53]. As a result, the independent traveller segment of the tourism sector who is more likely to visit rural and remote areas of the state has small sample sizes because the total number of visitors to these areas is small [23]. Thus, the independent traveller segment is underrepresented in AVSP hampering more comprehensive analysis of niche market development such as bird tourism. More in depth coverage of independent travellers would have been able to capture bird tourism spending in Alaska’s high-end birding destinations located in the Islands region [23,54,55].

To the contrary, the AVSP data does not distinguish between primary purpose birdwatching and incidental bird observing [6,21]. Since there was no such distinction, we attributed all spending by bird watching visitors to bird tourism, regardless of how many other activities birders engaged in and regardless of underlying unknown motivations to come to Alaska in the first place. Further research related to Alaska’s independent traveller segment, travelers’ behavior and motivations, and visitation to the remote rural parts of the state can provide more detailed insights for policy aimed at fostering sustainable tourism-related community development [22,23].

### Nature-based tourism and development

Historically, rural Alaska has had limited economic opportunities and high poverty rates [19]. It is also predominately inhabited by Alaska’s indigenous peoples who continue to have strong cultural and traditional ties to the land, practicing a subsistence lifestyle [24]. Our example of bird tourism provides a case study for how various regions across Alaska are taking advantage of nature-based tourism opportunities with the greatest potential for growth in the Arctic Plains, Western Alaska, and Islands regions.

Development and growth of the nature-based tourism sector can be fostered through sustainable infrastructure investments and borrowing strategies from other Alaska Regions. For example, the virtual Southeast Alaska Birding Trail links birding destinations along the Alaska Marine Highway by offering a virtual guide to birding hotspots across 18 remote Southeast Alaska communities [56]. The collection of sites mostly chosen by local birders along a pre-described route, is designed to maximize viewing opportunities for birders and to advance birding experience and visitor satisfaction [6,7,41]. We note, birding trails are not directly associated with physical built trail infrastructure but can utilize local trails that are already in place to facilitate access to birding hotspots. Given demand for bird watching grows, additional built infrastructure such as lodging and transportation infrastructure.

Further, developing niche tourism markets also diversifies the overall tourism sector, minimizes the effects of seasonality, and reduces visitor congestion elsewhere by distributing more visitors to remote regions that often are economically disadvantaged [41]. Local communities play a central role in driving nature-based tourism opportunities in these regions, making sure that outcomes are environmentally sensitive and culturally appropriate with a fair distribution of social, environmental, and economic benefits across local stakeholders [57,58]. Such approaches are known as regenerative tourism and go beyond sustainability by “contributing to the proactive regeneration of communities, cultures, heritage, places, and landscapes [57].” If co-designed with local communities, regenerative approaches to nature-based tourism development can lead to social and economic empowerment of indigenous communities and sustainable long-term outcomes [58,59].

### Nature-based tourism and biodiversity conservation

Nature-based tourism is also widely viewed as an important conservation tool for engaging and partnering with indigenous communities, especially for those who seek to diversify and grow their generally small economic base [9,18,60,61]. To reduce the current pace of global biodiversity loss, effective conservation efforts require a rapidly deployable all hands on deck approach that relies on indigenous knowledge and leadership from communities often inhabiting or owning critical conservation lands [62]. For birds and other migratory species especially, efficient conservation strategies require habitat connectivity and thus conservation action at large scale [63].

Globally, Alaska plays a significant role in such biodiversity conservation, due to its vast tracts of intact habitat supporting complete wildlife assemblages and the world’s largest concentration of shore birds [9]. In addition, Alaska’s indigenous population inhabits much of the wild lands required for achieving global biodiversity targets and is one of the largest landowners in Alaska. The Alaska Native Claims Settlement Act (ANCSA) established twelve Alaska Native regional corporations and over 200 local village corporations owning approximately 10% of Alaska’s land mass. Alaska is also home to 229 federally recognized tribes primarily inhabiting Alaska’s remote rural areas.

Since much of these areas are without protection, the support of private landowners for conservation will largely depend on the received financial benefits [64]. Effective conservation action to reduce biodiversity loss must retain the most-intact habitats and work more closely with rural communities [65]. Synergistic projects that tie conservation targets with nature-based tourism projects provide alternative income sources for rural communities and can be conservation-friendly alternatives without degrading natural capital compared to resource extraction, for example [66]. Indigenous place-based knowledge will be critical for co-designing projects where conservation outcomes are effective but also financially sustainable for communities [58,67].

## Conclusions

Birding tourism illustrates the growing and significant economic opportunities for nature-based tourism in rural and remote regions of Alaska. Most of Alaska’s tourism is focused on cruise ship destination tourism. Even though Alaska is home to more national park land than any other state, few national parks and other protected areas are visited because of their remoteness. Birding tourism illustrates one of many nature-based tourism activities that can inspire independent travellers to seek out new destinations in Alaska, expanding the economic footprint of tourism across the state.

Rural and remote regions of Alaska are predominately inhabited by indigenous peoples who have had limited economic opportunities but are surrounded by vast tracks of increasingly rare wildlife habitat of global significance. Our results showed that bird tourism provides critical cash flow and rare employment and income opportunities for local communities, presenting viable alternatives to resource extraction. The study discussed the need for partnerships with indigenous land holders centered around nature-based tourism opportunities tied to conservation for reducing global biodiversity loss. Alaska’s largely intact wilderness areas and indigenous land holders and federally recognized tribes play a critical role for global biodiversity conservation and improved stewardship of the globe’s wilderness areas.

## Supporting information

S1 Table Ebird data by census area and NABCI region. (DOCX)

S2 Table IMPLAN regions used in the input-output model corresponding to NABCI regions and census areas. (DOCX)

S3 Table Proportions allocating AVSP spending categories to IMPLAN sectors by regional model. (DOCX)

S4 Table Detailed input-output modelling results by region and impact type. (DOCX)

S5 Table Survey instrument. (PDF)

S1 Dataset (CSV)

S1 Code R-code used for AVSP data analysis and ebird data. (RMD)
