# Supplementary material for: Small sight—Big might: Economic impact of bird tourism shows opportunities for rural communities and biodiversity conservation
Source: PLoS One. 2022 Jul 6;17(7):e0268594. doi: 10.1371/journal.pone.0268594 (PMC9258859; doi:10.1371/journal.pone.0268594)
Supplement: S2 Table — (DOCX) [file pone.0268594.s002.docx]

Supplementary Information

Small sight – big might: Economic impact of bird tourism shows opportunities for rural communities and biodiversity conservation

Tobias Schwoerer and Natalie Dawson

**S3 Table. IMPLAN regions used in the input-output model corresponding to NABCI regions and census areas.**
