# Supplementary material for: Small sight—Big might: Economic impact of bird tourism shows opportunities for rural communities and biodiversity conservation
Source: PLoS One. 2022 Jul 6;17(7):e0268594. doi: 10.1371/journal.pone.0268594 (PMC9258859; doi:10.1371/journal.pone.0268594)
Supplement: S5 Table — (PDF) [file pone.0268594.s005.pdf]

Alaska Visitor Statistics Program 7

State of Alaska
Department of Commerce, Community,
& Economic Development

A. Interviewer Name: ____________________________ **B. Date:** ___________________________

C. Mode and Location

DOMESTIC AIR	INTERNATIONAL AIR	CRUISE SHIP	HIGHWAY ⇨	E. Vehicle Type
01❒ Anchorage	06❒ Anchorage	10❒ Ketchikan	11❒ Alcan	01❒ RV/camper
02❒ Fairbanks	07❒ Fairbanks		12❒ Taylor	02❒ Car/truck/van
03❒ Juneau	**FERRY**	⇩	13❒ Skagway	03❒ Motorcoach/bus
04❒ Ketchikan	08❒ to Bellingham		14❒ Haines	04❒ Motorcycle
05❒ Sitka ⇩	09❒ to Prince Rupert	**D. Ship Name**		05❒ Other ____________

F. Airline and Flight # ___________________________ **G. Refusals** _________________________

1. What was the main purpose for this trip? (Read 1-5)

1❒ Vacation/pleasure
2❒ Visiting friends or relatives
3❒ Business
4❒ Business and pleasure
5❒ Seasonal worker or student → **DISCONTINUE**
(seasonal work includes commercial fishing, guiding, oil field work)

2. Which state or country are you visiting from?

U.S. (2b)

01❒ Alabama	15❒ Iowa	28❒ Nevada	41❒ South Dakota	
03❒ Arizona	16❒ Kansas	29❒ New Hampshire	42❒ Tennessee	
04❒ Arkansas	17❒ Kentucky	30❒ New Jersey	43❒ Texas	
05❒ California	18❒ Louisiana	31❒ New Mexico	44❒ Utah	
06❒ Colorado	19❒ Maine	32❒ New York	45❒ Vermont	
07❒ Connecticut	20❒ Massachusetts	33❒ North Carolina	46❒ Virginia	
08❒ Delaware	21❒ Maryland	34❒ North Dakota	47❒ Washington	
09❒ Florida	22❒ Michigan	35❒ Ohio	48❒ Washington, D.C.	
10❒ Georgia	23❒ Minnesota	36❒ Oklahoma	49❒ West Virginia	
11❒ Hawaii	24❒ Mississippi	37❒ Oregon	50❒ Wisconsin	
12❒ Idaho	25❒ Missouri	38❒ Pennsylvania	51❒ Wyoming	
13❒ Illinois	26❒ Montana	39❒ Rhode Island		
14❒ Indiana	27❒ Nebraska	40❒ South Carolina		

Canada (2c) → **Which province or territory?**

01❒ Alberta
02❒ British Columbia
03❒ Manitoba
04❒ New Brunswick
05❒ Newfoundland/Labrador
06❒ Northwest Territories
07❒ Nova Scotia
08❒ Nunavut
09❒ Ontario
10❒ Prince Edward Island
11❒ Quebec
12❒ Saskatchewan
13❒ Yukon

International (2a)

01❒ Argentina	08❒ Denmark	14❒ Israel	20❒ New Zealand	26❒ Sweden
02❒ Australia	09❒ France	15❒ Japan	21❒ Norway	27❒ Taiwan
03❒ Austria	10❒ Germany	16❒ Korea	22❒ Portugal	28❒ Thailand
04❒ Belgium	11❒ India	17❒ Malaysia	23❒ Russia	29❒ United Kingdom
05❒ Brazil	12❒ Italy	18❒ Mexico	24❒ Spain	2aa❒ Other:
07❒ China	13❒ Ireland	19❒ Netherlands	25❒ Switzerland	

3. What mode of transportation did you use to enter Alaska?

1❒ Air
2❒ State ferry
5❒ Other ___________________
3❒ Highway ⇨
4❒ Cruise ship

3a. What type of vehicle were you using?

1❒ RV/Camper 2❒ Car/truck/van 3❒ Motorcoach/bus
4❒ Motorcycle 5❒ Other _________________________

4. Did you travel to more than one community while in Alaska? 1❒ Yes 2❒ No **(skip to Q5)**

4a. I'd like to ask you about the modes of transportation you used within Alaska, once you arrived. Which of the following modes of transportation did you use to travel between communities within Alaska? (Read 1-8)

01❒ Air	03❒ Alaska Railroad	05❒ Rental vehicle	07❒ Personal vehicle	09❒ None of the above
02❒ State ferry	04❒ Tour bus or van	06❒ Rental RV	08❒ Personal RV	10❒ Don't know/refused

5. Is this your first trip to Alaska? 1❒ Yes **(Skip to #8)** 2❒ No

6. Not including this trip, how many times have you been to Alaska for vacation? ___________

2❒ Used to live here 3❒ DK/Ref.

7. Before this trip, had you ever traveled by cruise ship in Alaska? 1❒ Yes 2❒ No

8. On this current trip, how many nights total were you in Alaska?
If you overnighted on a cruise ship or ferry, please include all nights on board. ___________

9a. Where in Alaska did you stay each night while on your trip? **(Show grid.)**
9b. How many nights and what type of lodging?

	Hotel/ motel	Lodge	B&B	Vacation rental	Friends/ Family	Campground/ RV	Wilderness Camping	Cruise Ship (9d)	State Ferry (9c)	Other	10. DAY VISIT ?
01❐ State ferry											
02❐ Cruise ship											
03❐ Other vessel											
04❐ Anchorage											04❐
05❐ Chicken											05❐
06❐ Coldfoot											06❐
07❐ Copper Center											07❐
08❐ Cordova											08❐
09❐ Delta Junction											09❐
10❐ Denali Nat'l Park											10❐
11❐ Fairbanks											11❐
12❐ Girdwood/Alyeska											12❐
13❐ Glacier Bay Nat'l Park											13❐
14❐ Glennallen											14❐
15❐ Gustavus											15❐
16❐ Haines											16❐
17❐ Healy											17❐
18❐ Homer											18❐
19❐ Hoonah/Icy Strait Pt											19❐
20❐ Juneau											20❐
21❐ Kenai/Soldotna											21❐
22❐ Kenai Peninsula: Cooper Landing / Hope / Ninilchik / Sterling											22❐
23❐ Ketchikan											23❐
24❐ Kodiak											24❐
25❐ Nome											25❐
26❐ Palmer/Wasilla											26❐
27❐ Petersburg											27❐
28❐ Portage											28❐
29❐ Prince of Wales Is.											29❐
30❐ Prince William Sound											30❐
31❐ Seward											31❐
32❐ Sitka											32❐
33❐ Skagway											33❐
34❐ Talkeetna											34❐
35❐ Tok											35❐
36❐ Valdez											36❐
37❐ Whittier											37❐
38❐ Wrangell											38❐
39❐ Other (1)											39❐
40❐ Other (2)											40❐
41❐ Other (3)											41❐
42❐ Other (4)											42❐

10. Did you visit any communities or destinations without spending the night? 44❐ None **OR**

NON-CRUISE VISITORS ONLY (INCLUDES AMHS)

11. Excluding transportation to and from Alaska, can you estimate how much your traveling party spent on your entire Alaska trip? Your traveling party are those with whom you shared expenses. $______________ 2❐ Don't know 3❐ Ref.

4❐ **CHECK HERE** if amount includes transportation because respondent was unable to separate transportation costs.

12. Did your party purchase any multi-day packages that included lodging, transportation, and activities?

1❐ Yes ⇒ **12a. Which of the following best describes this package?** (Read 1-6)

- 01❐ Fishing lodge package
- 02❐ Wilderness lodge package
- 03❐ Motorcoach tour
- 04❐ Rail package
- 05❐ Rental car or RV package
- 06❐ Adventure tour (hiking, biking, kayaking, rafting)
- 07❐ Other ______________________________

2❐ No
3❐ Don't know
4❐ Refused

12b. Which portions of your trip were included in this package? (Show list)

01❐ Air 03❐ Meals 05❐ Railroad 07❐ Vehicle/RV rental 09❐ Fishing
02❐ Lodging 04❐ Tours 06❐ Ferry 08❐ Bus/motorcoach 10❐ Other __________

12c. Can you estimate the price per person for this package? $______________ 2❐ Don't know 3❐ Ref.

4❐ **CHECK HERE** if amount applies to party.

STATE FERRY PASSENGERS ONLY

13. Can you estimate the price per person for your ferry tickets? $______________ 2❐ Don't know 3❐ Ref.

4❐ **CHECK HERE** if amount applies to party.

CRUISE VISITORS ONLY

14. What was the name of your cruise line? ______________________________ 2❐ Don't know 3❐ Ref.

15. Which of the following best describes your Alaska trip? (Read 1-4)

1❐ Round trip cruise from Vancouver, Seattle, or San Francisco **(skip to Q17)**
2❐ Cruise one-way, fly one-way
3❐ Cruise with an overnight Alaska land tour
4❐ In-state or small ship cruise
5❐ Other ______________

16. Did you spend any nights in Alaska on your own, in addition to your cruise or land tour package?

1❐ Yes 2❐ No 3❐ Don't know

17. Can you estimate the price per person for your cruise or cruise/tour package? $__________ 2❐ Don't know 3❐ Ref.

4❐ **CHECK HERE** if amount applies to party.

17a. Did this price include airfare? 1❐ Yes 2❐ No 3❐ Don't know 4❐ Ref.

18. Can you estimate what your traveling party spent on the rest of your Alaska trip? Please include tours your party bought before and during your trip. $________ 2❐ Don't know 3❐ Ref.

19. Now I'd like to ask you about your party's spending in each community. Let's start with (community). **While in** (community)**, about how much did your party spend on lodging?** Show categories.
If "none," enter $0. If "don't know," enter DK.

Include all communites from Q9/10	**Lodging**	**Tours Activities** Entertainment	**Gifts Souvenirs Clothing**	**Food Beverage**	**Rental Cars Fuel** Transportation	**Other** (inc. package if in 1 community)
**a.**	$	$	$	$	$	$
**b.**	$	$	$	$	$	$
**c.**	$	$	$	$	$	$
**d.**	$	$	$	$	$	$
**e.**	$	$	$	$	$	$
**f.**	$	$	$	$	$	$
**g.**	$	$	$	$	$	$
**h.**	$	$	$	$	$	$
**i.**	$	$	$	$	$	$
**j.**	$	$	$	$	$	$
**k.**	$	$	$	$	$	$
**l.**	$	$	$	$	$	$
**m.**	$	$	$	$	$	$
**n.**	$	$	$	$	$	$
**o.**	$	$	$	$	$	$

20. Which tours or activities did you participate in while in Alaska? (Show list)	21. Where did you participate in these activities?
01❒ ATV/4-wheeling/ORV/Jeep	
02❒ Biking	
03❒ Bird watching	
04❒ Business	
05❒ Camping	
06❒ City/sightseeing tours	
07❒ Day cruises whale watch, wildlife, glacier, riverboat, jet boat	
08❒ Dog sledding/kennel tour	
09❒ Fishing (guided)	
10❒ Fishing (unguided)	
11❒ Flightseeing	
12❒ Gold panning/mine tour	
13❒ Hiking/nature walk	
14❒ Historical/cultural attractions Pioneer Park, festivals, Russian history	
15❒ Hot springs	
16❒ Native cultural tours/activities	
17❒ Hunting	
18❒ Kayaking/canoeing	
19❒ Museums	
20❒ Northern Lights viewing	
21❒ Rafting	
22❒ Salmon bake/crab feed	
23❒ Shopping	
24❒ Shows/Alaska entertainment	
25❒ Train – Alaska Railroad	
26❒ Train – White Pass/Yukon	SKAGWAY
27❒ Tramway/gondola	
28❒ Wildlife viewing	
29❒ Zip-line	
30❒ Other activity:	
31❒ Other activity:	
32❒ Other activity:	

(SKIP Q22-Q23 FOR BUSINESS-ONLY TRAVELERS)

22. How well did your trip to Alaska live up to what you expected from an Alaska vacation? Was it… (Read 1-5)

1❒ Much higher than expectations 3❒ About what you expected 4❒ Below expectations 6❒ Don't know
2❒ Higher than expectations 5❒ Far below expectations 7❒ Refused

23. In terms of value for the money, how does Alaska compare with other vacation destinations you've visited in the past five years? Was it… (Read 1-5)

1❒ Much better 3❒ About the same 4❒ Worse 6❒ Don't know
2❒ Better 5❒ Much worse 7❒ Refused

24. How satisfied were you with your overall experience in Alaska? Were you… (Read 1,2,4,5)

1❒ Very satisfied 3❒ Neither/neutral 4❒ Dissatisfied 6❒ Don't know
2❒ Satisfied 5❒ Very dissatisfied 7❒ Refused

25. How likely are you to recommend Alaska as a vacation destination to others? (Read 1-4)

1❒ Very likely 3❒ Unlikely 5❒ Don't know
2❒ Likely 4❒ Very unlikely 6❒ Refused

26. How likely are you to return to Alaska in the next five years? (Read 1-4)

1❒ Very likely **(ASK 26a)** 3❒ Unlikely 5❒ Don't know
2❒ Likely 4❒ Very unlikely 6❒ Refused

26a. What are you most interested in experiencing on your next Alaska trip? (Check only one)

01❒ Arctic 05❒ Fishing 09❒ Hiking 13❒ Wildlife 16❒ Don't know
02❒ Camping 06❒ Flightseeing 10❒ Native culture 14❒ Winter 17❒ Refused
03❒ Cruise 07❒ Friends/family 11❒ Northern Lights 15❒ Other ______________________
04❒ Denali 08❒ Glaciers 12❒ Train

27. How far in advance did you decide to come on this trip to Alaska? ________# of months OR 2❒ < 1 month
3❒ Don't know

28. How far in advance did you book your major travel arrangements? ________# of months OR 2❒ < 1 month
3❒ Did not book anything 4❒ Don't know

29. Did you use the internet, including any apps, to plan or book any portion of your Alaska trip?
1❒ Yes 2❒ No **(Skip to Q34)** 3❒ Don't know **(Skip to Q34)**

30. Did you visit the official State of Alaska travel website travelalaska.com?
1❒ Yes 2❒ No 3❒ Don't know 4❒ Refused

31. Which other websites or apps did you use in planning your Alaska trip? (Show list) 1❒ Don't know **(Skip to Q34)**

32. Which of these sites did you use to book portions of your Alaska trip?
1❒ None/did not book online **(Skip to Q34)** 2❒ Don't know **(Skip to Q34)**

	31. Planned	32. Booked		31. Planned	32. Booked
a. AAA.com	1	2	q. Hotwire	1	2
b. AirBnB	1	2	r. Instagram	1	2
c. Airline websites	1	2	s. Kayak.com	1	2
d. Alaska Marine Highway	1	2	t. LonelyPlanet.com	1	2
e. Alaska App	1	2	u. Orbitz	1	2
f. Booking.com	1	2	v. Priceline	1	2
g. Car/RV rental websites	1	2	w. Tour company websites	1	2
h. CruiseCritic.com	1	2	x. Travelocity	1	2
i. Cruise line websites	1	2	y. Trip Advisor	1	2
j. Expedia	1	2	z. Twitter	1	2
k. Facebook	1	2	aa. Visitors Bureaus	1	2
l. Google	1	2	bb. VRBO	1	2
m. Hipmunk	1	2	cc. Yelp	1	2
n. HotelTonight	1	2	dd. Other ________________	1	2
o. Hotels.com	1	2	dd. Other ________________	1	2
p. Hotel/lodging websites	1	2	dd. Other ________________	1	2

33. Which portions of your trip did you book online, including through apps?
01❒ Airfare 03❒ Tours 05❒ Cruise 07❒ Overnight packages 09❒ DK/R
02❒ Lodging 04❒ Vehicle rental 06❒ Ferry 08❒ Other ________________________

34. Did you book any portion of your trip through a travel agent?

1❒ Yes → **34a. Which portions of your trip did you book through a travel agent?**
2❒ No
3❒ Don't know
4❒ Refused

01❒ Airfare 03❒ Tours 05❒ Cruise 07❒ Overnight packages 09❒ DK/R
02❒ Lodging 04❒ Vehicle rental 06❒ Ferry 08❒ Other ________________

35. Did you receive the State of Alaska Official Vacation Planner?

1❒ Yes 2❒ No 3❒ Don't know 4❒ Refused

36. What other sources did you use to get information about Alaska not including online sources? (Show list)

01❒ AAA
02❒ Brochures
03❒ Club/organization/church
04❒ Community brochures
05❒ Convention & Visitor Bureau(s)
06❒ Cruise line
07❒ Ferry brochure/schedule
08❒ Friends/family/co-workers
09❒ Hotel/lodge
10❒ Library
11❒ Magazine
12❒ Newspaper
13❒ Prior experience
14❒ Television
15❒ Tour company
16❒ Travel/recreation exhibitions
17❒ *Milepost*
18❒ *North to Alaska* Guide
19❒ Other travel guide/book
20❒ Other
21❒ Other
22❒ **None**
23❒ Don't know/refused

37. Including yourself, how many people are traveling in your immediate party?
By party, I mean those sharing expenses such as food, lodging, and transportation. __________

38. Including yourself, what is the total number of people traveling in your group?
By group, I mean friends or relatives that are traveling with you. __________

39. What are the ages and gender of the ______ people in your traveling party? (Enter first 8 members of party only)

	39a.		39b.			39a.		39b.
	**Male**	**Female**	**Age**			**Male**	**Female**	**Age**
Yourself #1	1	2			Traveler #5	1	2	
Traveler #2	1	2			Traveler #6	1	2	
Traveler #3	1	2			Traveler #7	1	2	
Traveler #4	1	2			Traveler #8	1	2	

40. Which of these party members did most of the planning for this Alaska trip? Traveler # ______ 1❒ DK/Ref.

41. Are there children living in your household? 1❒ Yes 2❒ No 3❒ Don't know 4❒ Refused

42. Are you retired, or semi-retired? 1❒ Yes 2❒ No 3❒ Don't know 4❒ Refused

43. Please point to the highest level of education you had the opportunity to complete.

01❒ Grade 11 or less
02❒ High school graduate/GED
03❒ Associate/technical degree
04❒ Some college
05❒ Graduated from college
06❒ Masters/Doctorate

07❒ Don't know
08❒ Other ____________________________
09❒ Refused

44. Please point to the category that best describes your household. (Before-tax income, US$)

01❒ Less than $25,000
02❒ $25,000 to $50,000
03❒ $50,000 to $75,000
04❒ $75,000 to $100,000
05❒ $100,000 to $125,000
06❒ $125,000 to $150,000
07❒ $150,000 to $200,000
08❒ More than $200,000
❒ Other currency: ________

09❒ Don't know
10❒ Refused

45. The State of Alaska may wish to contact you in the future to find out more about your Alaska experiences. Would you be willing to provide your email address? Your information would not be shared or used for any purposes other than visitor research.

01❒ Yes ____ 02❒ No 03❒ Don't have email

Thank you!
